# Circulating Concentrations of Vitamin B6 and Kidney Cancer Prognosis: A Prospective Case-Cohort Study

**DOI:** 10.1371/journal.pone.0140677

**Published:** 2015-10-27

**Authors:** David C. Muller, Mattias Johansson, David Zaridze, Anush Moukeria, Vladimir Janout, Ivana Holcatova, Marie Navratilova, Dana Mates, Øivind Midttun, Per Magne Ueland, Paul Brennan, Ghislaine Scelo

**Affiliations:** 1 International Agency for Research on Cancer (IARC), 150 cours Albert Thomas, 69372 Lyon cedex 08, France; 2 Russian N.N. Blokhin Cancer Research Centre, Kashirskoye Shosse 24, 115478 Moscow, Russian Federation; 3 Department of Preventive Medicine, Faculty of Medicine, Palacky University, Hnevotinska 3, CZ 775 15 Olomouc, Czech Republic; 4 Institute of Hygiene and Epidemiology, First Faculty of Medicine, Charles University Prague, Studnickova 7, CZ 128 00 Prague 2, Czech Republic; 5 Department of Cancer Epidemiology and Genetics, Masaryk Memorial Cancer Institute, Zluty kopec 7, CZ 656 53, Brno, Czech Republic; 6 National Institute of Public Health, Str. Dr. Leonte Anastasievici Nr.1-3, Sector 5, 050463 Bucharest, Romania; 7 Bevital AS, co/Helse Bergen, Laboratoriebygget 9 etg., NO-5021 Bergen, Norway; 8 Department of Clinical Science, University of Bergen, New Laboratory Building, 9th floor, NO-5021 Bergen, Norway; 9 Laboratory of Clinical Biochemistry, Haukeland University Hospital, P.O. Box 1400, 5021 Bergen, Norway; Institute of Bioengineering and Nanotechnology, SINGAPORE

## Abstract

Prospective cohort studies have found that prediagnostic circulating vitamin B6 is inversely associated with both risk of kidney cancer and kidney cancer prognosis. We investigated whether circulating concentrations of vitamin B6 at kidney cancer diagnosis are associated with risk of death using a case-cohort study of 630 renal cell carcinoma (RCC) patients. Blood was collected at the time of diagnosis, and vitamin B6 concentrations were quantified using LC-MS/MS. Hazard ratios (HR) and 95% confidence intervals (CI) were calculated using Cox regression models. After adjusting for stage, age, and sex, the hazard was 3 times lower among those in the highest compared to the lowest fourth of B6 concentration (HR_4vs1_ 0.33, 95% CI [0.18, 0.60]). This inverse association was solely driven by death from RCC (HR_4vs1_ 0.22, 95% CI [0.11, 0.46]), and not death from other causes (HR_4vs1_ 0.89, 95% CI [0.35, 2.28], p-interaction = 0.008). These results suggest that circulating vitamin B6 could provide additional prognostic information for kidney cancer patients beyond that afforded by tumour stage.

## Background

Each year more than 300,000 new cases of kidney cancer are diagnosed worldwide, leading to approximately 130,000 deaths [1]. The prognosis is strongly dependent on stage at diagnosis, with around 90% of stage I patients alive five years after diagnosis, compared with only 10% of stage IV patients [2].

Kidney cancer rates are approximately two times higher for males than females, and increase with age. In addition to these factors, hypertension, obesity and tobacco smoking are the main established risk factors for kidney cancer [3]. Little is known on factors influencing the survival after diagnosis, apart from tumor stage and grade. Some studies have suggested a favorable prognosis for obese or overweight patients, which is surprising as obesity is also associated with an increased risk of disease onset. Whether overweight and obese individuals present with more indolent tumors or whether they benefit from earlier detection with fortuitous diagnosis in the course of frequent medical examinations is unclear [4–7]. We recently investigated biomarkers of one-carbon metabolism and kidney cancer onset and survival in two prospective epidemiological cohorts, where blood samples were collected at entry to the studies, an average of 7 years before diagnosis [8]. We reported a strong inverse relationship between levels of circulating vitamin B6 and the risk of subsequent kidney cancer, as well as the risk of death after kidney diagnosis. Although our analysis suggested that this association was driven by deaths caused by kidney cancer, available data did not allow for a thorough cause-specific mortality analysis, nor could we assess the importance of concentrations measured at diagnosis. Data were also scarce on important prognostic factors such as stage. This observation prompted us to investigate whether vitamin B6 concentrations at diagnosis of kidney cancer are indicative of subsequent survival, and if they are informative beyond stage or other prognostic factors.

## Materials and Methods

### The K2 study

Participants included patients who were above 18 years of age and diagnosed with kidney cancer between 2007 and 2012 in one of 6 participating centres in Czech Republic, 1 center in Romania, and 1 center in Russia. We gave participants a standardized face-to-face short lifestyle questionnaire covering socio-demographic characteristics, anthropometric measures, medical history, family history, and tobacco and alcohol use. Clinical and pathological data were abstracted from medical charts and pathological reports. A majority of participants underwent nephrectomy and the tumor was histologically confirmed. Follow-up for outcome (relapse, vital status, and cause of death where relevant) was performed every 6 to 12 months after diagnosis, using passive follow-up methods where possible (with confirmation of vital status through active follow-up methods in case of uncertainties), and active follow-up methods when no linkage to databases was possible. The study protocol was approved by the institutional review boards of the International Agency for Research on Cancer (reference 09–24) and all collaborating institutions, and we obtained written informed consent from all participants.

### Case-cohort sampling

The case-cohort is an efficient design that allows unbiased estimation of relative risks without the need to ascertain covariate information for an entire cohort [9]. A case-cohort sample consists of a—possibly stratified—random sample of all participants in the cohort, as well as all participants who experienced the event of interest during follow-up, but were not randomly selected for inclusion in the subcohort. Subsequent analyses are weighted to account for the unequal probability of inclusion in the sample. In the present study, the “cohort” was the entire sample of renal cell carcinoma (RCC) cases, and the event of interest was death from any cause.

Among 2330 participants with questionnaire data available and a diagnosis of RCC, we excluded 125 participants with no plasma sample available, 1005 participants with no follow-up data at the time of this project, 5 participants with inconsistencies in reference dates, and 7 participants with no information on stage. From the 1188 remaining, we randomly selected 500 participants at baseline (the subcohort). We also included all participants who died during follow-up that were not randomly selected in to the subcohort (*N* = 93), as well as all eligible stage IV patients (*N* = 37) that had survived and were not randomly selected. Hence a total of 630 participants diagnosed with kidney cancer were included in the study. Within the randomly selected subcohort, the median follow-up time was 2.6 years (minimum 5 days, maximum 6.7 years).

### Biosample processing and biochemical analysis

Venous blood was obtained before or at the time of the nephrectomy, prior to any treatment. Blood was collected in vacutainers containing ethylenediaminetetraacetic acid (EDTA), and processed as rapidly as possible (usually within two hours). Plasma samples were stored at −80°C, except in Ceske Budejovice, Czech Republic, where samples were stored at −20°C. All samples were transported at −80°C to IARC for long-term storage at −150°C. Samples underwent a single thawing cycle for aliquoting of 400uL for shipment to the Bevital laboratory in Bergen, Norway, for analysis. Vitamin B6 (measured as pyridoxal 5’-phosphate, its active form) was quantified using liquid chromatography coupled to tandem mass spectrometry. Details and performance of this method have been previously published [10]. Notably it has been shown that storage conditions and duration do not lead to substantial degradation of vitamin B6, and that technical variation between batches is acceptably small [10].

### Statistical analysis

We used Cox proportional hazards models with time since diagnosis (recruitment) as the time scale to estimate hazard ratios (HR) and 95% confidence intervals (CI) for all-cause mortality by categories of circulating vitamin B6. Categories were defined by splitting B6 concentrations at quartiles of its distribution within the randomly selected subcohort. HRs for cause-specific mortality were calculated in a competing risks model using the data augmentation method of Lunn and McNeil [11]. To account for the case-cohort design [9] we used Barlow’s method to weight the likelihood and computed robust variance estimates, slightly adjusted to account for the fact that we included all cases with stage IV disease (i.e., all stage IV cases received a weight of 1). All models included stage, age at diagnosis, and sex as covariates, with the baseline hazard stratified by country of recruitment. We additionally adjusted for body mass index (BMI, kg/m^2^), smoking status (never, former, current), cigarettes per day, alcohol drinking status (never, former, current), and alcohol intake (ml/day of ethanol). We also conducted a sensitivity analysis adjusting for Fuhrman grade (with participants with missing grade excluded), and receipt of secondary treatment.

We investigated potential effect modification by fitting interactions between log_2_ transformed vitamin B6 concentrations and age categories, country of recruitment, sex, stage, histological type, history of diabetes, history of hypertension, smoking status, alcohol intake status, and categories of BMI. Model based estimates of the survival function by categories of B6 concentration and stage were obtained from flexible parametric survival models [12], using the same weighting for the likelihood as applied to the Cox models. Restricted cubic splines with 2 knots (placed at the 33rd and 67th percentiles of the uncensored log survival times) were used to model the baseline cumulative hazard.

In a secondary analysis, we used standard, unweighted Cox regression to compute HRs for the joint outcome of relapse (local or systemic) or death, whichever occurred first, as well as a competing risks analysis of relapse versus death. These analyses excluded stage IV cases (who are not disease-free after diagnosis and thus cannot relapse by definition) and those who died who had not been randomly sampled for the subcohort, as the case-cohort sample did not include the corresponding participants from outside the subcohort who relapsed.

All *p*-values are two sided, and were calculated using the Wald test. Statistical analyses were performed using Stata 12.1 for Linux (Stata Corporation, College Station, Texas, US) and R version 3.1.0 [13].

## Results

Demographic and clinical characteristics of the study sample by vital status at the end of follow-up are presented in Table 1. The sample included a higher proportion of men (63%) than women, and were predominantly recruited from the Czech Republic (52%) and Russia (43%), with only 5% of participants recruited in Romania. Those participants who survived until the end of follow-up had a similar age distribution to those who died during follow-up. 518 of the 630 cases (82%) were conventional RCC. 50% of deaths occurred among participants with a stage IV tumor, and 15% of those surviving to the end of follow-up had been diagnosed with stage IV disease. In contrast, 72% of those surviving to the end of follow-up were diagnosed with stage I-II disease.

**Table 1:** Demographic and clinical characteristics of the participants by vital status at the end of follow-up.

		Vital status				
		alive		dead		
		n	(%)	n	(%)	Total
	Total	427	(100)	203	(100)	630
Sex	Male	266	(62)	131	(65)	397
	Female	161	(38)	72	(35)	233
Age at recruitment (years)	[26.7,55)	118	(28)	37	(18)	155
	[55,65)	161	(38)	88	(43)	249
	[65,75)	115	(27)	52	(26)	167
	[75,86.8]	33	(8)	26	(13)	59
Country	Czech Republic	230	(54)	95	(47)	325
	Russia	167	(39)	105	(52)	272
	Romania	30	(7)	3	(1)	33
BMI (kg/m^2^)	[17.2,25)	97	(23)	71	(35)	168
	[25,30)	184	(43)	82	(40)	266
	[30,58.5]	143	(33)	50	(25)	193
	missing	3	(1)	0	(0)	3
smoking	Never smoker	213	(50)	92	(45)	305
	Former smoker	108	(25)	54	(27)	162
	Current smoker	106	(25)	57	(28)	163
Alcohol	Never drinker	266	(62)	118	(58)	384
	Former drinker	38	(9)	33	(16)	71
	Current drinker	123	(29)	51	(25)	174
	missing	0	(0)	1	(0)	1
Diabetic	Yes	65	(15)	39	(19)	104
	No	362 (100)	(85)	164	(81)	526
Hypertension	Yes	233	(55)	103	(51)	336
	No	193	(45)	100	(49)	293
	missing	1	(0)	0	(0)	1
Stage	I	269	(63)	38	(19)	307
	II	38	(9)	10	(5)	48
	III	56	(13)	54	(27)	110
	IV	63	(15)	101	(50)	164
	missing	1	(0)	0	(0)	1
Grade	1	73	(17)	7	(3)	80
	2	173	(41)	53	(26)	226
	3	67	(16)	50	(25)	117
	4	15	(4)	18	(9)	33
	missing	99	(23)	75	(37)	174
Histology	Conventional RCC	357	(84)	161	(79)	518
	Papillary RCC	41	(10)	10	(5)	51
	Chromophobe RCC	11	(3)	5	(2)	16
	Other	14	(3)	4	(2)	18
	Unknown	4	(1)	23	(11)	27
Received secondary treatment	No	357	(84)	123	(61)	480
	Yes	70	(16)	80	(39)	150
Circulating B6 (nmol/L)	1 [2.6,19.8)	83	(19)	100	(49)	183
	2 [19.8,32.7)	115	(27)	52	(26)	167
	3 [32.7,49.4)	110	(26)	30	(15)	140
	4 [49.4,467.5]	119	(28)	21	(10)	140

Hazard of death from any cause was strongly and inversely associated with circulating concentrations of vitamin B6 (Table 2). After adjusting for stage, age, and sex, the hazard was 3 times lower among those in the highest compared to the lowest fourth of B6 concentration (HR_4vs1_ 0.33, 95% CI [0.18, 0.60]). A competing risks analysis suggested that this inverse association was solely driven by death from RCC (HR_4vs1_ 0.22, 95% CI [0.11, 0.46]), and not death from other causes (HR_4vs1_ 0.89, 95% CI [0.35, 2.28], *p*-interaction = 0.008). Further adjustment for BMI, smoking status, cigarettes per day, alcohol drinking status, and mL of alcohol per day did not affect the estimates (Table 2). Considering relapse or death as a joint outcome, or as separate outcomes in a competing risks analysis, yielded essentially identical estimates to the analysis of all-cause mortality (see S1 Table), as did models that were additionally adjusted for grade (see S2 Table), or receipt of any secondary treatment (see S3 Table).

**Table 2:** Hazard ratios (HR) [95% confidence intervals (CI)] for risk of all cause and cause specific mortality by categories of circulating vitamin B6 concentration.

			minimally adjusted^†^				adjusted^‡^			
Cause of death	B6 group^#^	*N*_deaths_	HR	[95% CI]	*p* *	*p*_het_ ^§^	HR	[95% CI]	*p* *	*p*_het_ ^§^
all cause	1	100	1.00		.000014		1.00		.000057	
	2	52	0.74	[0.46, 1.18]			0.75	[0.46, 1.23]		
	3	30	0.47	[0.27, 0.80]			0.47	[0.27, 0.84]		
	4	21	0.33	[0.18, 0.60]			0.34	[0.18, 0.63]		
RCC	1	86	1.00			.0078	1.00			.016
	2	40	0.70	[0.42, 1.15]			0.70	[0.42, 1.18]		
	3	15	0.29	[0.15, 0.55]			0.30	[0.15, 0.58]		
	4	11	0.22	[0.11, 0.46]			0.24	[0.11, 0.50]		
non-RCC	1	14	1.00				1.00			
	2	12	1.02	[0.44, 2.39]			1.06	[0.45, 2.50]		
	3	15	1.41	[0.61, 3.25]			1.40	[0.59, 3.31]		
	4	10	0.89	[0.35, 2.28]			0.85	[0.31, 2.31]		

^#^Groups were defined as follows: 1 [2.6, 19.8), 2 [19.8, 32.7], 3 [32.7, 49.4), 4 [49.4, 467.5] nmol/L

^†^Stratified by country, and adjusted for stage, age at recruitment, and sex

^‡^Additionally adjusted for BMI (kg/m^2^), smoking status, cigarettes per day, alcohol drinking status, and ethanol intake per day (mL)

*p-values for the all-cause models are from tests against the null hypothesis that the vitamin B6 coefficients are identically 0 (test with 3 degrees of freedom).

^§^ *p*_het_-values for the competing risks model are from tests against the null hypothesis of no heterogeneity of the coefficients by cause of death (RCC versus non-RCC, test with 3 degrees of freedom).

**Fig 1:**
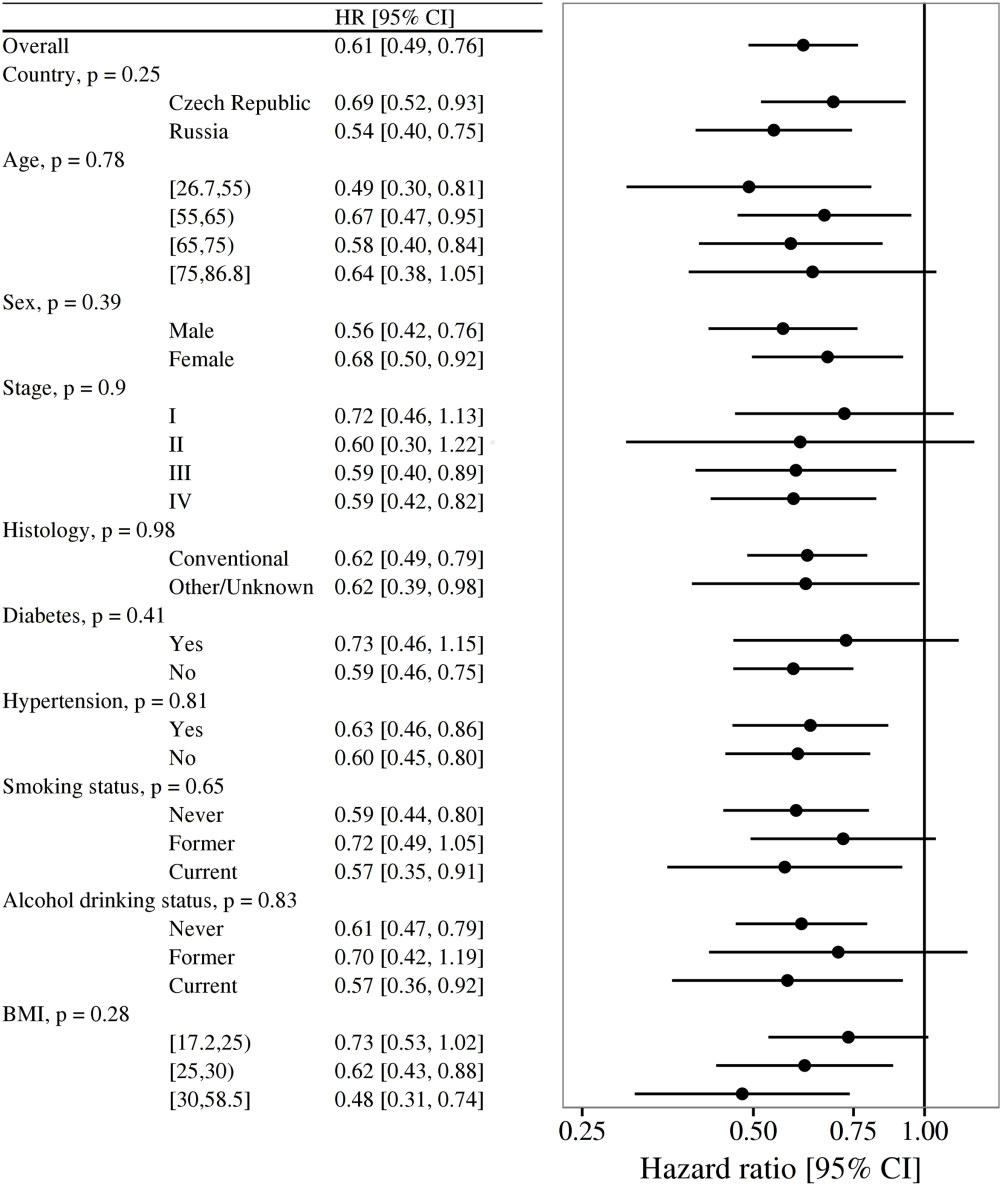
Hazard ratios (HR) and 95% confidence intervals (CI) for a doubling in vitamin B6 concentration by potential effect modifiers. Estimates derived from Cox models stratified by country of recruitment, and adjusted for stage, age at recruitment, and sex. *p*-values are from Wald tests of the interaction terms. A separate estimate for Romania is not provided due to an insufficient number of observations.

Fig 1 presents HRs for a doubling in B6 concentration separately by categories of several potential effect modifiers. The estimated magnitude of the association was consistent by age, sex, stage, histology, history of diabetes and hypertension, smoking status, and alcohol intake status. There was some indication that the association might be stronger among those with BMI ≥ 30, but there was no statistical evidence of interaction (HR 0.48, 95% CI [0.31, 0.74] for BMI ≥ 30 versus 0.73 (0.53 to 1.02) for BMI < 25; *p*-interaction = 0.28). While BMI itself was inversely associated with hazard of death (HR for BMI ≥ 30 versus < 25 0.53, 95% CI [0.34, 0.83]), this association was attenuated somewhat by adjustment for stage (HR 0.68, 95% CI [0.43, 1.10]), and further attenuated after additionally adjusting for both stage and categories of circulating B6 (HR 0.76, 95% CI [0.46, 1.26]).

**Fig 2:**
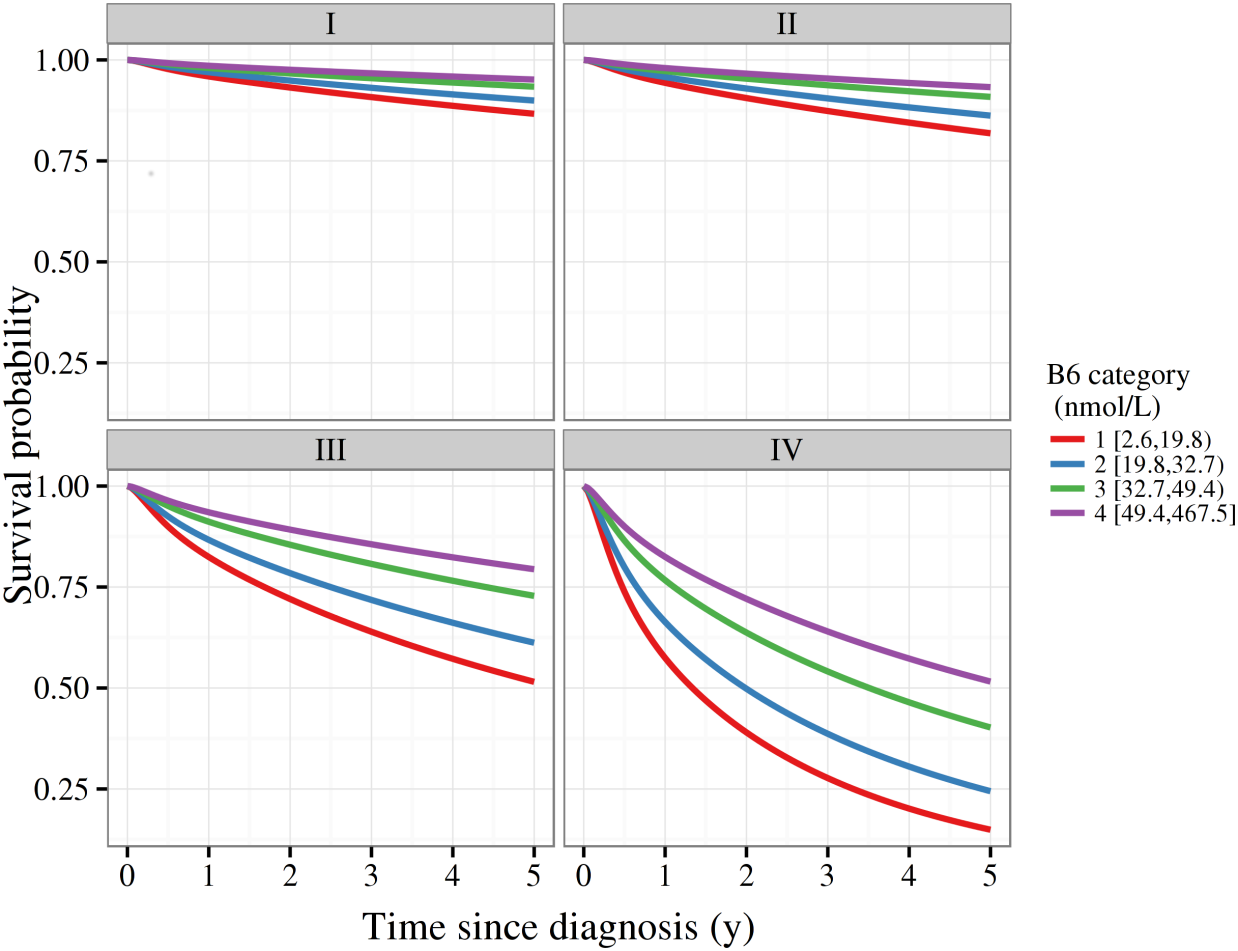
Model based survival function by categories of circulating vitamin B6 and stage. Estimates derived from flexible parametric survival models assuming proportional hazards for B6 categories, no interaction between stage and B6 categories, and a diagnosis age of 60y.

Model based survival functions given diagnosis at age 60 years by category of B6 concentrations and stage are plotted in Fig 2. For any given stage, survival is discernibly poorer with lower circulating B6. For instance, given a stage I diagnosis at age 60 years, we estimate the 5-year survival probability to be 0.95 [95% CI 0.92, 0.97] for the highest category of concentration, versus 0.87 (0.79 to 0.92) for the lowest. Similarly, given a stage IV diagnosis the 5-year survival probability is 0.52 (0.31 to 0.69) for the highest category, and 0.15 (0.07 to 0.26) for the lowest.

## Discussion

We investigated whether differences in circulating concentrations of vitamin B6 at the time of diagnosis of RCC were associated with all-cause, cause-specific, and relapse-free survival. We observed that higher concentrations of vitamin B6 were associated with a substantially lower risk of death, in particular RCC specific death. Notably, we estimated 5-year survival to be 52% for stage IV cases in the highest B6 category, and 15% in the lowest.

This investigation was motivated by our recent studies of pre-diagnostic biomarkers of one-carbon metabolism and risk of RCC in prospective cohorts [8]. In both the European Prospective Investigation into Cancer and Nutrition (EPIC) and Melbourne Collaborative Cohort Study (MCCS) cohorts there was evidence of a strong inverse association between circulating concentration of vitamin B6 and risk of RCC. Additionally, pre-diagnostic circulating vitamin B6 was inversely associated with hazard of death among RCC cases in both the EPIC and MCCS cohorts. In EPIC, the HR for the highest versus the lowest fourth of concentration was 0.57 (95% CI [0.37, 0.87]). Although there was limited information available on cause of death and disease stage, the association was slightly stronger among those known to have died from kidney cancer, and was attenuated somewhat when adjusting for stage. Our results are consistent with these initial findings, and provide further evidence that a real association exists between vitamin B6 concentration at diagnosis and subsequent survival from RCC. Notably, our results also demonstrate that the association is likely to be much stronger for B6 concentrations at diagnosis than it is for prediagnostic concentrations. Additionally, our results suggest that this association is restricted to death from RCC specifically rather than death from any other cause, and is not wholly accounted for by differences in stage.

Little is known regarding factors influencing survival after RCC diagnosis. Although obesity is a strong risk factor for RCC, with each 5 kg/m^2^ increment in BMI associated with an increase in risk of approximately 30% [14], several studies have suggested that obesity might be associated with better prognosis. For instance, Choi et al. meta-analysed data from 15 prospective studies and found that obese patients had improved overall survival [4]. Ohno et al. also found an inverse association, and reported that the association persisted among males after adjustment for stage [6]. In contrast, among 2119 RCC patients at Memorial Sloan Kettering Cancer Center, the observed association between BMI and cause-specific mortality (HR for obese versus normal BMI 0.59, 95% CI [0.42, 0.83]) was attenuated after adjustment for stage and grade (HR 0.75, 95% CI [0.53, 1.07]) [7]. Our data corroborate this result, suggesting that any association between BMI and risk of death is not independent of stage.

In addition to stage attenuating the association between BMI and risk of death, we observed further attenuation after adjustment for circulating vitamin B6. This raises the intriguing possibility that the observed association with BMI is due to BMI acting as a crude marker of nutritional status. Indeed, composite indices of nutritional status or deficiency have been shown to associate with RCC prognosis. An index incorporating low BMI, low albumin, and high pre-operative weight loss was associated with almost 3-fold higher rate of cause-specific mortality among 369 patients with RCC at the Vanderbilt University Medical Center [15]. Similarly, an index of nutritional deficiency including low BMI, low albumin, and low cholesterol has been associated with approximately 2-fold higher rate of RCC-specific death [16]. Our observation that the association with BMI is almost entirely attenuated after adjustment for vitamin B6, and the indication that the association between vitamin B6 and survival might be strongest for those with high BMI, lend further and direct support to the hypothesis that nutritional status may be an important prognostic factor for RCC.

It is notable that the distribution of vitamin B6 concentrations in our sample is low compared to the general population. In healthy participants from Norway who had vitamin B6 assayed using non-fasting blood samples and the same platform employed for the present study, the median concentration was 48 nmol/L [17]. In contrast, we observed a median of 33 nmol/L in the subcohort of our study. A concentration of 30 nmol/L has been suggested as a lower bound for normal concentration [18], and concentrations less than 20 nmol/L have been considered deficient [19]. By these criteria, over 50% of our sample have concentrations below the normal range, and 30% would be considered deficient. While it is tempting to infer that dietary intervention or supplementation might lead to improved prognosis, this inference is somewhat premature, as vitamin B6 is associated with numerous pathways implicated in cancer development and progression, including the tryptophan metabolism pathway which is involved in immune function and inflammatory processes [20–22]. Similarly, vitamin B6 concentrations are correlated with albumin [23], which has also been shown to be inversely associated with risk of RCC specific death [7, 16, 24–26]. Thus our results should not be taken as evidence of a direct causal association between vitamin B6 concentration and prognosis.

One limitation of our study is that grade was unavailable for a substantial proportion (28%) of participants. Nevertheless, sensitivity analyses adjusting for grade and excluding those with missing data yielded substantively identical results, suggesting that the association between vitamin B6 and risk of death is not explained by grade. Another limitation is that we were unable to assess whether vitamin B6 is associated with prognosis independently of albumin, and thus cannot attempt to disentangle whether vitamin B6 per-se confers a survival advantage. It is also possible that these results may not generalise beyond Central and Eastern European populations, but the remarkable consistency with results observed in Western European cohort studies using pre-diagnostic blood samples would suggest that that our results will generalise well, at least to a broader population of European origin.

In summary, aside from tumour stage, little is known about factors associated with kidney cancer prognosis. We provide strong evidence that low circulating concentrations of vitamin B6 at diagnosis are associated with an increased risk of death from kidney cancer, and that circulating vitamin B6 provides additional prognostic information for kidney cancer patients beyond that afforded by tumour stage. In summary, higher circulating concentrations of vitamin B6 at the time of RCC diagnosis are strongly associated with better survival, independently of disease stage.

## Supporting Information

S1 Table Hazard ratios (HR) [95% confidence intervals (CI)] for risk of relapse free survival, and from a competing risks model of relapse versus death, by categories of circulating vitamin B6 concentration, for stage I-III. (PDF)

S2 Table Hazard ratios (HR) [95% confidence intervals (CI)] for risk of all cause and cause specific mortality by categories of circulating vitamin B6 concentration, additionally adjusting for grade (participants with missing grade excluded). (PDF)

S3 Table Hazard ratios^†^ (HR) [95% confidence intervals (CI)] for risk of all cause and cause specific mortality by categories of circulating vitamin B6 concentration, additionally adjusting for receipt of secondary treatment. (PDF)

S4 Table Demographic and clinical characteristics of the participants for those included and not included in the case-cohort sample. (PDF)

S1 File Data for the K2 case-cohort study of circulating vitamin B6 and RCC prognosis. (CSV)
