# Supplementary material for: Circulating Concentrations of Vitamin B6 and Kidney Cancer Prognosis: A Prospective Case-Cohort Study
Source: PLoS One. 2015 Oct 27;10(10):e0140677. doi: 10.1371/journal.pone.0140677 (PMC4623509; doi:10.1371/journal.pone.0140677)
Supplement: S1 Table — (PDF) [file pone.0140677.s001.pdf]

Supplementary Table 1: Hazard ratios (HR) [95% confidence intervals (CI)] for risk of relapse free survival, and from a competing risks model of relapse versus death, by categories of circulating vitamin B6 concentration, for stage I-III cases.

			minimally adjusted†				adjusted‡			
Type of event	B6 group#	$N^{¶}_{\text{events}}$	HR	[95% CI]	p^*	$p^{§}_{\text{het}}$	HR	[95% CI]	$p*$	$p^{§}_{\text{het}}$
Relapse or Death	1	32	1.00		.0041		1.00		.0061	
	2	12	0.22	[0.11, 0.43]			0.21	[0.10, 0.42]		
	3	17	0.48	[0.26, 0.91]			0.54	[0.28, 1.04]		
	4	14	0.34	[0.18, 0.67]			0.33	[0.17, 0.66]		
Relapse	1	19	1.00			.85	1.00			.87
	2	8	0.26	[0.11, 0.61]			0.26	[0.11, 0.61]		
	3	9	0.46	[0.20, 1.06]			0.51	[0.22, 1.21]		
	4	7	0.31	[0.13, 0.76]			0.32	[0.13, 0.80]		
Death	1	13	1.00				1.00			
	2	4	0.16	[0.05, 0.51]			0.15	[0.05, 0.49]		
	3	8	0.50	[0.20, 1.27]			0.56	[0.22, 1.45]		
	4	7	0.39	[0.15, 1.00]			0.34	[0.13, 0.94]		

#Groups were defined as follows: 1 [2.6, 19.8), 2 [19.8, 32.7], 3 [32.7, 49.4), 4 [49.4, 467.5] nmol/L

¶Number of deaths and/or relapses occurring among the stage I-III cases within the randomly selected subcohort. Deaths occurring outside the subcohort are not included in this analysis, nor are stage IV cases who by definition cannot relapse, so there are fewer events than in the main analysis presented in Table 2.

†Stratified by country, and adjusted for stage, age at recruitment, and sex

‡Additionally adjusted for BMI (kg/m^2), smoking status, cigarettes per day, alcohol drinking status, and ethanol intake per day (mL)

*p-values for the relapse free models are from tests against the null hypothesis that the vitamin B6 coefficients are identically 0 (test with 3 degrees of freedom).

§p_{het}-values for the competing risks model are from tests against the null hypothesis of no difference between the coefficients for relapse versus death (test with 3 degrees of freedom).
