# Supplementary material for: Circulating Concentrations of Vitamin B6 and Kidney Cancer Prognosis: A Prospective Case-Cohort Study
Source: PLoS One. 2015 Oct 27;10(10):e0140677. doi: 10.1371/journal.pone.0140677 (PMC4623509; doi:10.1371/journal.pone.0140677)
Supplement: S2 Table — (PDF) [file pone.0140677.s002.pdf]

Supplementary Table 2: Hazard ratios† (HR) [95% confidence intervals (CI)] for risk of all cause and cause specific mortality by categories of circulating vitamin B6 concentration, additionally adjusting for grade (participants with missing grade excluded).

Cause of death	B6 group#	N_{deaths}	HR	[95% CI]	p^*	$p^{§}_{het}$
all cause	1	54	1.00		.026	
	2	34	1.01	[0.55, 1.88]		
	3	20	0.68	[0.32, 1.43]		
	4	18	0.50	[0.23, 1.08]		
RCC	1	45	1.00			.062
	2	24	0.89	[0.46, 1.73]		
	3	10	0.41	[0.17, 1.00]		
	4	9	0.31	[0.12, 0.79]		
non-RCC	1	9	1.00			
	2	10	1.60	[0.56, 4.58]		
	3	10	1.91	[0.65, 5.66]		
	4	9	1.38	[0.44, 4.36]		

#Groups were defined as follows: 1 [2.6, 19.8), 2 [19.8, 32.7], 3 [32.7, 49.4), 4 [49.4, 467.5] nmol/L

†Stratified by country, and adjusted for stage, age at recruitment, sex, BMI (kg/m^2), smoking status, cigarettes per day, alcohol drinking status, ethanol intake per day (mL), and grade (with participants with missing grade excluded).

* p-values for the all cause models are from tests against the null hypothesis that the vitamin B6 coefficients are identically 0 (test with 3 degrees of freedom).

§ p_{het}-values for the competing risks model are from tests against the null hypothesis of no heterogeneity of the coefficients by cause of death (RCC versus non-RCC, test with 3 degrees of freedom).
