# Supplementary material for: Circulating Concentrations of Vitamin B6 and Kidney Cancer Prognosis: A Prospective Case-Cohort Study
Source: PLoS One. 2015 Oct 27;10(10):e0140677. doi: 10.1371/journal.pone.0140677 (PMC4623509; doi:10.1371/journal.pone.0140677)
Supplement: S3 Table — (PDF) [file pone.0140677.s003.pdf]

Supplementary Table 3: Hazard ratios[†] (HR) [95% confidence intervals (CI)] for risk of all cause and cause specific mortality by categories of circulating vitamin B6 concentration, additionally adjusting for receipt of secondary treatment.

Cause of death	B6 group[#]	N_{deaths}	HR	[95% CI]	p^*	$p_{\text{het}}^{\S}$
all cause	1	100	1.00		.000056	
	2	51	0.75	[0.45, 1.23]		
	3	30	0.47	[0.27, 0.85]		
	4	20	0.34	[0.18, 0.63]		
RCC	1	45	1.00			.016
	2	24	0.70	[0.41, 1.19]		
	3	10	0.30	[0.15, 0.58]		
	4	9	0.24	[0.11, 0.50]		
non-RCC	1	9	1.00			
	2	10	1.05	[0.44, 2.51]		
	3	10	1.39	[0.59, 3.31]		
	4	9	0.85	[0.31, 2.31]		

[#]Groups were defined as follows: 1 [2.6, 19.8), 2 [19.8, 32.7], 3 [32.7, 49.4), 4 [49.4, 467.5] nmol/L

[†]Stratified by country, and adjusted for stage, age at recruitment, sex, BMI (kg/m^2), smoking status, cigarettes per day, alcohol drinking status, ethanol intake per day (mL), and receipt of secondary treatment.

[*] p-values for the all cause models are from tests against the null hypothesis that the vitamin B6 coefficients are identically 0 (test with 3 degrees of freedom).

[§] p_{het}-values for the competing risks model are from tests against the null hypothesis of no heterogeneity of the coefficients by cause of death (RCC versus non-RCC, test with 3 degrees of freedom).
