# Supplementary material for: Circulating Concentrations of Vitamin B6 and Kidney Cancer Prognosis: A Prospective Case-Cohort Study
Source: PLoS One. 2015 Oct 27;10(10):e0140677. doi: 10.1371/journal.pone.0140677 (PMC4623509; doi:10.1371/journal.pone.0140677)
Supplement: S4 Table — (PDF) [file pone.0140677.s004.pdf]

Supplementary Table 4: Demographic and clinical characteristics of the participants for those included and not included in the case-cohort sample

		Included in study			
		No		Yes	
		n	(%)	n	(%)
	Total	1575	(100)	630	(100)
Sex	Male	968	(61)	397	(63)
	Female	607	(39)	233	(37)
Age at recruitment (years)	[17.6,55)	493	(31)	155	(25)
	[55,65)	584	(37)	249	(40)
	[65,86.8]	497	(32)	226	(36)
	missing	1	(0)	0	(0)
Country	Czech	568	(36)	325	(52)
	Romani	144	(9)	272	(43)
	Russia	797	(51)	33	(5)
	Serbia	66	(4)	0	(0)
BMI (kg/m^2)	[14.9,25)	386	(25)	168	(27)
	[25,30)	601	(38)	266	(42)
	[30,62.9]	582	(37)	193	(31)
	missing	6	(0)	3	(0)
Smoking	Never smoker	842	(53)	305	(48)
	Former smoker	341	(22)	162	(26)
	Current smoker	388	(25)	163	(26)
	missing	4	(0)	0	(0)
Diabetes	Yes	193	(12)	104	(17)
	No	1379	(88)	526	(83)
	missing	3	(0)	0	(0)
Hypertension	Yes	820	(52)	336	(53)
	No	752	(48)	293	(47)
	missing	3	(0)	1	(0)
Stage	I	718	(46)	307	(49)
	II	132	(8)	48	(8)
	III	255	(16)	110	(17)
	IV	175	(11)	164	(26)
	missing	295	(19)	1	(0)
Grade	1	214	(14)	80	(13)
	2	659	(42)	226	(36)
	3	256	(16)	117	(19)
	4	37	(2)	33	(5)
	9	406	(26)	174	(28)
	missing	3	(0)	0	(0)
Histology	Conventional RCC	1260	(80)	518	(82)
	Papillary RCC	129	(8)	51	(8)
	Chromophobe RCC	54	(3)	16	(3)
	Other	64	(4)	18	(3)
	Unknown	65	(4)	27	(4)
	missing	3	(0)	0	(0)
